# Plant Organelle DNA Maintenance

**DOI:** 10.3390/plants9060683

**Published:** 2020-05-28

**Authors:** Niaz Ahmad, Brent L. Nielsen

**Affiliations:** 1 Agricultural Biotechnology Division, National Institute for Biotechnology & Genetic Engineering, Jhang Road, Faisalabad 38000, Pakistan; niazbloch@yahoo.com; 2 Department of Microbiology & Molecular Biology, Brigham Young University, Provo, UT 84602, USA

**Keywords:** organelles, plastid phylogenetics, DNA replication, DNA recombination, plant organelle genome structure

## Abstract

Plant cells contain two double membrane bound organelles, plastids and mitochondria, that contain their own genomes. There is a very large variation in the sizes of mitochondrial genomes in higher plants, while the plastid genome remains relatively uniform across different species. One of the curious features of the organelle DNA is that it exists in a high copy number per mitochondria or chloroplast, which varies greatly in different tissues during plant development. The variations in copy number, morphology and genomic content reflect the diversity in organelle functions. The link between the metabolic needs of a cell and the capacity of mitochondria and chloroplasts to fulfill this demand is thought to act as a selective force on the number of organelles and genome copies per organelle. However, it is not yet clear how the activities of mitochondria and chloroplasts are coordinated in response to cellular and environmental cues. The relationship between genome copy number variation and the mechanism(s) by which the genomes are maintained through different developmental stages are yet to be fully understood. This Special Issue has several contributions that address current knowledge of higher plant organelle DNA. Here we briefly introduce these articles that discuss the importance of different aspects of the organelle genome in higher plants.

## 1. Introduction

Mitochondrial and chloroplast genomes have been studied for nearly 60 years, and much is known about the structure, gene content, expression and other characteristics of organelle genomes. Plant mitochondria, in great contrast to mitochondria in other organisms, have a very wide range in genome size and appear to be predominantly linear in structure. Chloroplast genomes are generally more consistent in size and may exist in both circular and linear forms [1]. Major questions that have persisted, however, relate to how these plant organelle genomes are replicated, repaired and maintained.

The endosymbiotic origin of mitochondria and chloroplasts has long been accepted, suggesting that the DNA replication mechanisms would be similar to their bacterial ancestors. However, none of the DNA replication, DNA recombination or DNA repair proteins required for maintenance of the organelle genomes are encoded in either of these organelle genomes [1,2]. The genes for these proteins are nuclear encoded and the protein products are imported into the proper organelle for function. The analysis of the minimal replisome in plant mitochondria and chloroplasts to date indicates that it is phage-like, most similar to the bacteriophage T7 system [1,2].

Plant organelle genomes—both mitochondrial and plastid—are relatively small (though larger than animal mitochondrial genomes) and are found in variable and sometimes incredibly high copy numbers. Their copy number varies from tissue to tissue, depending mainly on the age and type of the tissue. While higher plant mitochondria show a considerable degree of variation in their genome sizes, plastid genome size remains fairly uniform among different species [1].

Organellar genome substitution rates are much lower than the nuclear genome due to homologous recombination, which allows these molecules to be useful as molecular clocks to carry out evolutionary studies and determine genetic relationships among different taxa. In addition to phylogenetic studies, mitochondrial genomes provide excellent tools to gain insights into cytoplasmic male sterility (CMS) sources in plants.

Organelle genome stability is an absolute requirement for normal cell growth and function, and for proliferation. Faithful replication and repair of organellar genomes are therefore necessary to avoid genome instability, which may result in detrimental phenotypes. The mechanisms by which the organelle genomes in different tissues are replicated, maintained and repaired are not fully understood. This Special Issue was hosted to address unanswered questions related to organelle genome maintenance in plants. The Special Issue has eight helpful contributions that have attempted to answer some of these questions. The first four articles deal with questions regarding the maintenance and replication of the organelle genomes, whilst the last four articles deal with organellar genome sequencing and structural analyses.

## 2. Key Messages

### 2.1. Mechanisms for Plant Organelle Genome Replication and Repair

Brieba [2] summarizes the current literature to point out the importance of homologous DNA recombination in maintaining the integrity of plant mitochondrial genomes. Plant mitochondrial DNA (mtDNA) appears to be predominantly linear in nature and lacks any clearly identified replication origins. DNA replication proteins functional in both mitochondria and chloroplasts have been identified, and surprisingly, there are multiple copies of the genes for most of these proteins [1,2]. Other evidence in the literature suggests the presence of more than one mechanism for the replication of each of the plant organelle genomes [1,2]. These possibilities include typical primer-driven replication from distinct origins, recombination-dependent replication (RDR) and rolling circle replication.

Each of these mechanisms requires several common replication functions, but it is unclear how priming of DNA replication occurs in plant organelles. Twinkle is a DNA primase/helicase that is localized to both organelles in plants. In animal cells, this protein is absolutely required, but plant mutants are fully viable with no apparent differences compared with wild-type plants [1,2]. These two contributions discuss these observations and the possibility of other proteins being responsible for priming in plant organelles.

Plant organelle genomes, in particular those in mitochondria, contain repeat sequences that facilitate intramolecular recombination to generate various sub-genomic molecules. Recombination across these repeats, however, is regulated and suppressed by surveillance mechanisms including MSH1 and some Rec proteins [2,3]. MSH1 mutants in *Psychomytrella* show increased recombination across intermediate sized repeats relative to the wild-type [3].

Mutations in plant mitochondrial gene coding regions are rare, suggesting efficient repair of miss-paired or damaged bases such as uracil generated by deamination of cytidine. Proteins for mismatch and nucleotide excision repair have not been found in plant mitochondria [4], but base excision and double-strand break repair appear to be functional in both plant organelles. In Wynn et al. [4], uracil N-glycosylase (UNG) mutants were examined to determine if this defect in base excision repair (BER) would lead to more mutations, but this was not found. They did find that several genes that encode proteins involved in double-strand break repair (DSBR) were upregulated in the UNG mutants. This led to the conclusion that most damage to plant mitochondrial DNA is repaired by DSBR with further contribution by BER [4]. This implication of a major role for DSBR, which utilizes homologous DNA recombination, is supported by many studies that report the adverse effects of mutations in DNA recombination proteins on plant organelle integrity [1,3].

### 2.2. Characteristics and Rearrangements of Plant Organelle Genome Structure

Our knowledge about the genetic diversity of plant organelle genomes is currently based on small intergenic polymorphism regions. These same regions are used to conduct evolutionary studies and construct phylogenetic trees. However, internal transcribed spacer (ITS)-based trees do not have power to resolve the phylogenetic relationships among species and groups. With advances in sequencing technologies, organellar genomics is now moving away from individual gene analysis to whole genomes for the reconstruction of phylogenies at lower taxonomic levels, to study divergence among different groups, and for the structural analysis of these genomes. The availability of complete organellar genome sequences allows the construction of “species trees’” by which the accuracy of the phylogenetic relationships is greatly increased. Although gene-based phylogenetic trees, commonly referred to as “gene trees” could be readily obtained previously, they fail to trace all of the evolutionary events since not all genes evolve in a similar manner in the same lineage. Whole genome sequencing (WGS) provides valuable information about the occurrence of nucleotide recombination events in the organelle genomes.

The genus *Mammillaria* is an important genus due to its highly rich genetic diversity. Several of its species have been included in the Red List of Threatened Species of the International Union for Conservation of Nature. However, molecular marker-based phylogenetic studies fail to resolve the relationships among species of the *Mammillaria* genus. Solórzano et al. [5] obtained the complete chloroplast genome (plastome) sequences of seven different species of the short-globose cacti of *Mammillaria* and used a species tree to resolve the species of this genus.

The genus *Lilium* is widely distributed in the cold and temperate regions of the Northern Hemisphere. Many attempts using molecular phylogenetic approaches have been made; however, there remain unresolved clades in the genus. Kim et al. [6] sequenced the chloroplast genome of 28 *Lilium* species and used these sequences to construct a species tree in order to increase the accuracy of phylogenetic relationships for unresolved taxa.

WGS data also facilitate studies on the mechanisms of gene loss, recombination events, transfer of genes to the nucleus and evolution of different traits in different lineages. Cytoplasmic male sterility (CMS) has been extensively studied in sunflower (*Helianthus annuus* L.), which has more than 70 sources. This earlier work was primarily done by restriction fragment length polymorphism (RFLP) analysis, which does not provide a detailed explanation of the underlying events. One of the spontaneously occurring CMS sources in sunflower is ANN2, which is quite complicated and cannot be restored completely. Makarenko et al. [7] used the WGS method to gain insights into the structural reorganization of the mitochondrial genome that generates ANN2 in sunflower. The authors compared the mitochondrial genome sequences from a male sterile line with that of a male-fertile line and observed several reorganization events (deletions and insertions) and several new transcriptionally active open reading frames (ORFs) in the mitochondrial genome of the male sterile line. The deletion events resulted in the partial removal of the *atp6* (*orf1197*) gene and complete elimination of *orf777*, indicating a major role of the *atp6* gene in ANN2-type CMS.

It is now well-established that chloroplast genomes evolved from free-living cyanobacterial cells. The endosymbiotic events resulting in the conversion of these cells into organelles were accompanied by a massive transfer of their genetic material to the host cell nucleus, with only a small set of essential genes retained in their genomes. In heterotrophic plant species, with the loss of selection pressure over photosynthesis-related genes, this genome shrinkage is even higher. The plastome of such plant species provides an excellent tool to study the loss of organelle genetic material to the nucleus while they shift from the autotrophic to the heterotrophic mode. Jost et al. [8] sequenced the plastome of a holoparasitic plant species, *Prosopanche americana*, which has only 24 housekeeping genes and has lost the inverted repeats (IR) that are thought to stabilize the genome. The genetic machinery of *P. americana* shows an incredibly high degree of bias towards “AT”-rich codons, with >90% of the plastome sequence containing “A” and “T” nucleotides.

## 3. Future Directions

Considerable progress has been made on sequencing and characterizing plant organelle genomes since they were discovered about sixty years ago. However, there are numerous aspects of their replication and maintenance that are still unclear. Further work is needed to determine whether there is a single mechanism for DNA replication in plant mitochondria and chloroplasts. If there is more than one mechanism, what are they and when/how do they function? Do these mechanisms function at different stages of plant growth or in response to specific signals? It is still unclear what mechanisms control the initiation of plant organelle genome replication and copy number. What determines and regulates genome copy number in meristem and mature plant tissues? Why does genome copy number vary so widely in different tissues and ages of plants? These are important questions that require further research.

While DNA replication origins have been characterized in chloroplasts, there is as yet no conclusive evidence for distinct replication origins in plant mitochondria. This is a challenge that has been studied but no clear conclusions have been published. New approaches are needed to address this question. Along with this, it is unclear how plant organelle DNA replication is primed. Since Twinkle mutants are viable, there must be a separate mechanism for generating primers unless they are not needed (i.e., if homologous strand invasion provides the needed 3’ ends for DNA synthesis). If this is the case, this would suggest that homologous DNA recombination is a major contributor to genome replication in addition to its likely role in DNA repair. The role of DNA recombination in the replication and maintenance of plant organelle genomes thus deserves further study.

Regarding repair of DNA damage, the current literature suggests that double-stranded break repair and base excision repair may be the only mechanisms that function in plant organelles. If so, what are the relative contributions of each process in these organelles? Does nonhomologous end joining occur in plant organelles? And how does it affect genome integrity?

Finally, there are still questions regarding the evolution and variation of plant organelle genomes. Evolutionary adaptations may play a central role in plant mitochondrial genome size variation. However, it is unclear what mechanisms and selection processes determine plant organelle genome size. In addition, the mechanisms involved in purifying the selection by which parasitic plants keep some house-keeping genes, while losing other genes, and the IRs are unclear. Does this reduction in content affect genome stability? This is a promising area of research, and we look forward to new studies in the future to address these many questions.
